# Expression of the transcription factor, TFII-I, during post-implantation mouse embryonic development

**DOI:** 10.1186/1756-0500-3-203

**Published:** 2010-07-20

**Authors:** Iwona Fijalkowska, Deva Sharma, Carol J Bult, Sonye K Danoff

**Affiliations:** 1 Johns Hopkins University School of Medicine, Department of Medicine, Cardiopulmonary and Critical Care Division, 1830 E. Monument Street, Baltimore, MD 21205, USA; 2 The Jackson Laboratory, 600 Main Street, Bar Harbor, ME 04609, USA

## Abstract

**Background:**

General transcription factor (TFII-I) is a multi-functional transcription factor encoded by the Gtf2i gene, that has been demonstrated to regulate transcription of genes critical for development. Because of the broad range of genes regulated by TFII-I as well as its potential role in a significant neuro-developmental disorder, developing a comprehensive expression profile is critical to the study of this transcription factor. We sought to define the timing and pattern of expression of TFII-I in post-implantation embryos at a time during which many putative TFII-I target genes are expressed.

**Findings:**

Antibodies to the N-terminus of TFII-I were used to probe embryonic mouse sections. TFII-I protein is widely expressed in the developing embryo. TFII-I is expressed throughout the period from E8-E16. However, within this period there are striking shifts in localization from cytoplasmic predominant to nuclear. TFII-I expression varies in both a spatial and temporal fashion. There is extensive expression in neural precursors at E8. This expression persists at later stages. TFII-I is expressed in developing lung, heart and gut structures. There is no evidence of isoform specific expression. Available data regarding expression patterns at both an RNA and protein level throughout development are also comprehensively reviewed.

**Conclusions:**

Our immunohistochemical studies of the temporal and spatial expression patterns of TFII-I in mouse embryonic sections are consistent with the hypothesis that hemizygous deletion of *GTF2I *in individuals with Williams-Beuren Syndrome contributes to the distinct cognitive and physiological symptoms associated with the disorder.

## Background

TFII-I, or General Transcription Factor II-I (GTF2-I) is a member of ubiquitously expressed, multifunctional transcription factor family that integrates signals from multiple pathways and mediates cellular response to changes in the external environment [1]. Both the high degree of sequence conservation in TFII-I among species and the lack of individuals with homozygous deletion of *GTF2I *suggest that its ubiquitous expression and various molecular functions are essential for viability.

TFII-I gene (Entrez Gene ID 2969) has been mapped to an interval of the human chromosome 7q11.23 (chr7:73,805,362-73,812,956). This region is commonly deleted in Williams-Beuren syndrome (WBS) (OMIM#194050), which is typically associated with hemizygous microdeletion of a 1.6 Mb region containing about 16 genes [2-5]. In mouse, the gene (Entrez Gene ID 14886, MGI 1202722) is located on chromosome 5qG2 (chr5:134,713,704-134,790,616). Experimentally generated mutant mice heterozygotic for *Gtf2i *and for its related transcription factor *Gtf21-rd1 *featured anomalies similar to those observed in Williams-Beuren Syndrome: retarded growth, microcephaly and craniofacial and skeletal defects. Homozygous loss of G*tf2i *caused embryonic lethality [6].

Structurally, the TFII-I protein comprises several domains that define its biological function. Additional File 1 (Structure, Functions and Cellular Localization of TFII-I) presents schematic diagram of TFII-I protein structure and discusses functions of the domains. So far, direct involvement of TFII-I in gene regulation has been confirmed for more than 20 genes. Additional File 2 (TFII-I Target Genes) presents a list of genes that contain TFII-I binding sites in their sequences and that were found controlled by TFII-I.

In order to expand on the available description of timing and location of the expression of TFII-I documented in the literature [2,7,8] we reviewed a number of resources on gene expression profile. Additional File 3 (Developmental Expression of TFII-I mRNA and Reported Expression of TFII-I in Mouse Development) summarizes developmental expression of TFII-I mRNA based on profiles of expressed sequence tags (ESTs) available from NCBI [9], data from The Jackson Laboratory on Mouse Gene Expression Database [10,11] and EMAGE--Edinburgh Mouse Atlas of Gene Expression [12].

We wished to expand on the existing data regarding expression with a focus on protein expression using immunohistochemistry to provide spatial detail. Detailed methods that were applied are described in Additional File 4 (Methods).

## Findings

### Expression of TFII-I by developmental stage

**Figure 1:**
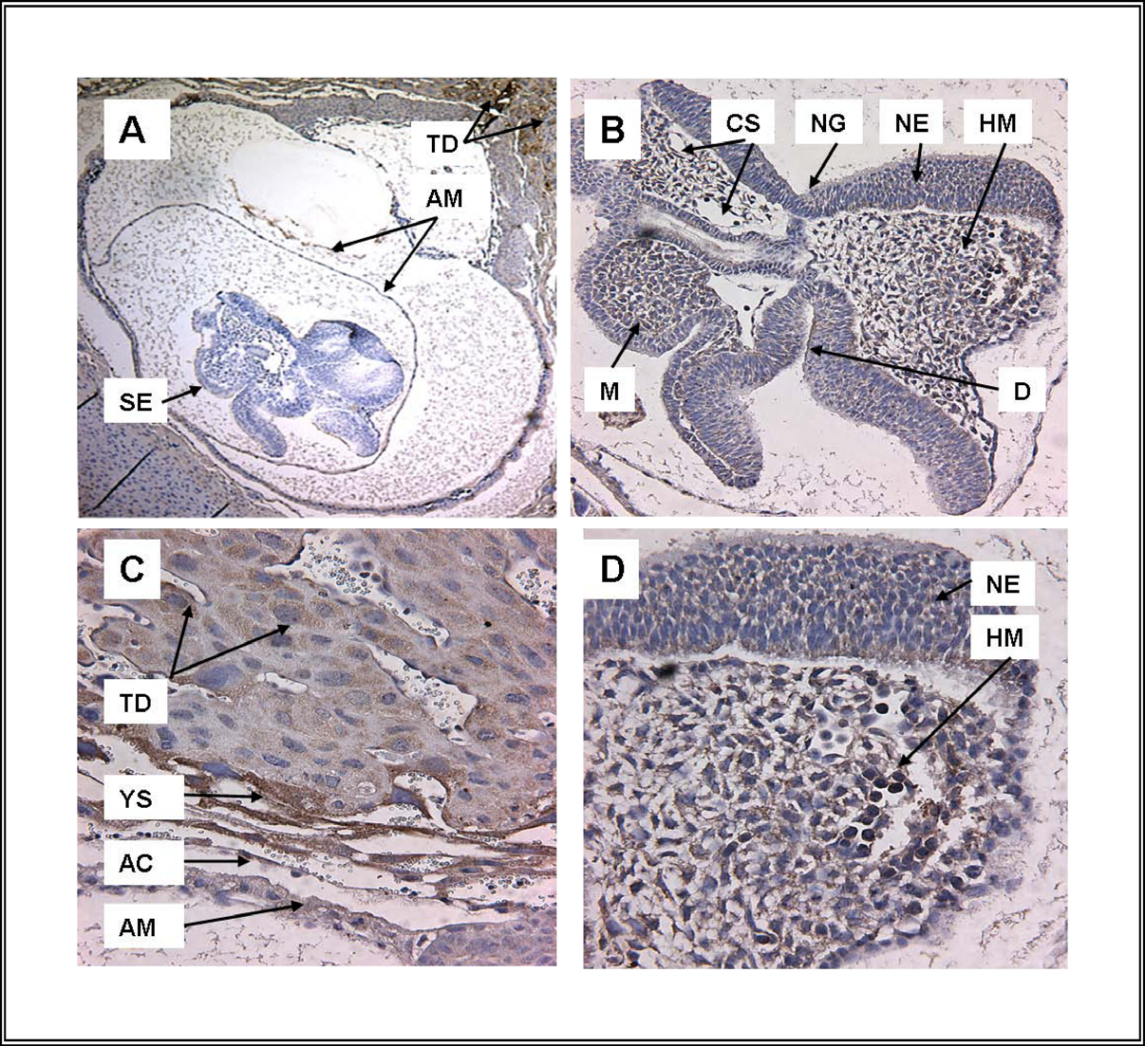
**FTFII-I expression at E8**. **A**. Embryo and placenta (5 ×), **B**. Embryo at ED8, head region (16 ×), **C**. Placenta at ED8 (32 ×), **D. **Embryo, brain region (32×). TFII-I immunoreactivity is present in both embryo and placenta at ED8. A and B. Extra-embryonic membranes demonstrate extensive TFII-I immunoreactivity in trophectoderm (TR) derivatives, as well as in yolk sac (YS) and amniotic membrane (AM). C and D. Within the embryo, neural ectoderm (NE) as well as mesoderm (M) shows TFII-I immunoreactivity. Of note, much of the immunoreactivity appears cytoplasmic in localization. Abbr.: AC-Amniotic Cavity, AM-Amniotic Membrane, CS-Cardio-vascular System, D-Prospective Diencephalon, HM-Head Mesenchyme, M-Mesoderm, NE-Neural Ectoderm, NG-Neural Groove, SE-Surface Ectoderm, TD-Trophoectoderm Derivatives, YS-Yolk Sac.

**Figure 2:**
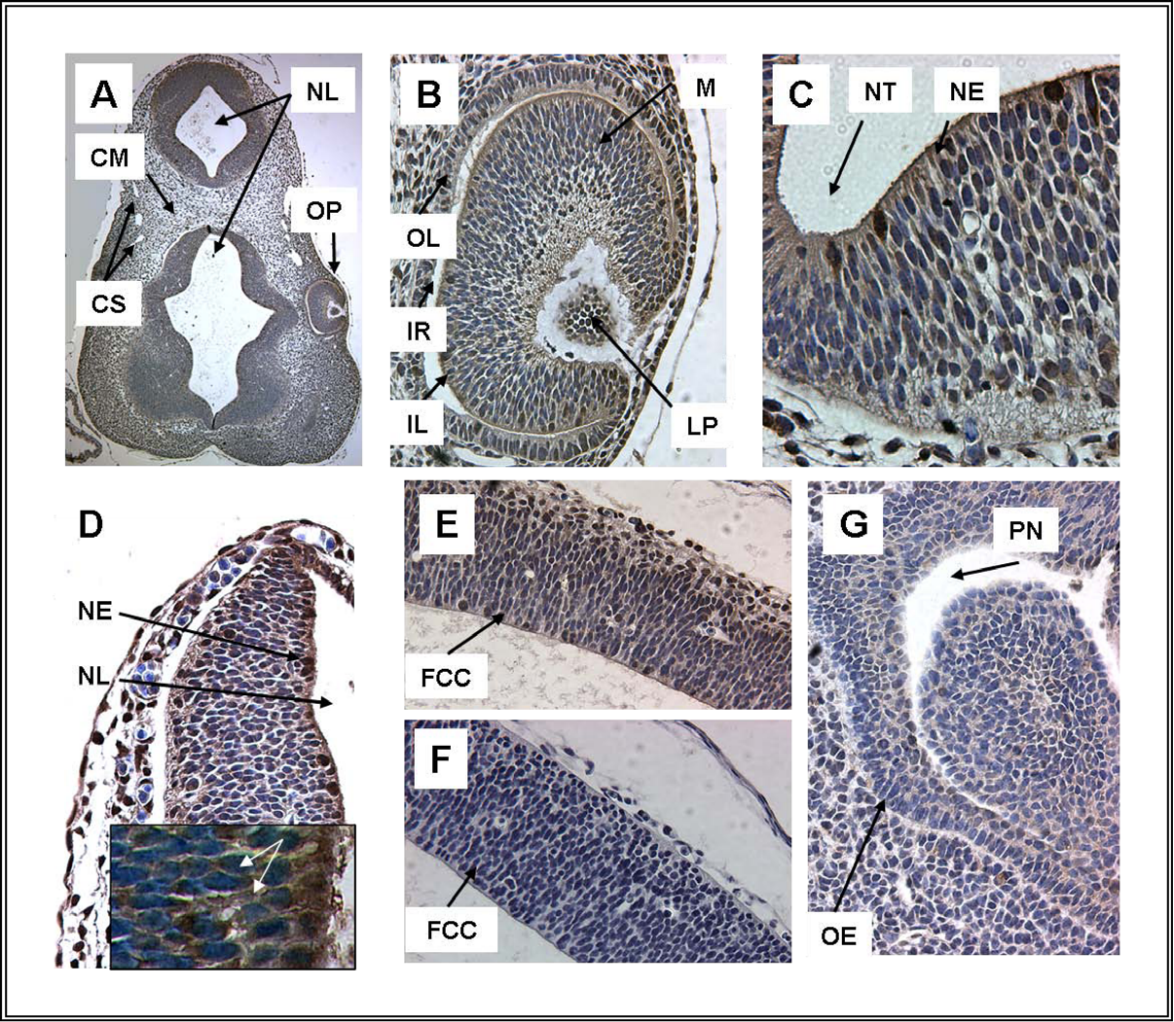
**TFII-I expression in head region at E9 (A-D) and at E12 (E, F, G)**. A. Head region (5 ×), B. Optic vesicle (32 ×), C. Neural tube (64 ×), D. Brain region (40 ×), E-F. Brain Region (32 ×), G. Nasopharynx (40 ×). TFII-I is more discretely localized at ED9 with nuclear localization. A. At low power, TFII-I immunoreactivity is visible in multiple regions of the embryo, but is most intense in the neural tube. B. The developing eye demonstrates TFII-I expression in the inner and outer retinal layer as well as lens primordium. C and D. Neural tube demonstrates intense nuclear immunoreactivity in individual cells. At E12 TFII-I is expressed extensively in brain, in contrast to TFII-IRD1, whereas TFII-I is only moderately present in nasopharynx. E. TFII-I is expressed in both nuclear and cytoplasmic localization in brain (32 ×). F. TFII-IRD1 antibody demonstrates no immunoreactivity in an adjacent section. G. Nasopharynx also demonstrates predominantly cytoplasmic localization (40 ×). Inset in Figure 2D shows nuclear and cytoplasmic locatization of TFII-I under 100 × magnification (Zeiss Axioscop, obj. Apochromat 100 ×). White arrows indicate positive reaction of specific antibody with TFII-I. Abbr.: CM-Cephalic Mesenchyme, CS-Cardiovascular System, FCC - Future Cerebral Cortex, IL-Inner Retinal Layer, IR-Intra-Retinal Space, LP-Lens Primordium, M-Mesenchyme, NE-Neuroepithelium, NL-Neural Lumen, NT-Neural Tube, OE-Olfactory Epithelium, OL-Outer Retinal Layer, OP-Optic Pit, PN- Primitive Nasopharynx,

As suggested by previous studies, TFII-I immunoreactivity is present in mouse embryo continuously from E8-E16. The intensity, distribution and subcellular localization vary, dependent on the stage. At E8 (Figure 1A-D), TFII-I is expressed at higher levels in trophectodermal derivatives rather than in the embryo. Within the embryo TFII-I in the ectoderm is largely cytoplasmic. Expression is also present in the mesoderm, predominantly in a cytoplasmic distribution. In contrast, at E9, TFII-I shows discrete nuclear localization in neuroectoderm in addition to a more diffuse cytoplasmic distribution (Figure 2A-D and the inlet). Extensive TFII-I immunoreactivity is also present in the eye primordium (Figure 2B).

With increasing organ differentiation at E11, TFII-I can be localized to a number of developing organ systems (noted in detail below).

**Figure 3:**
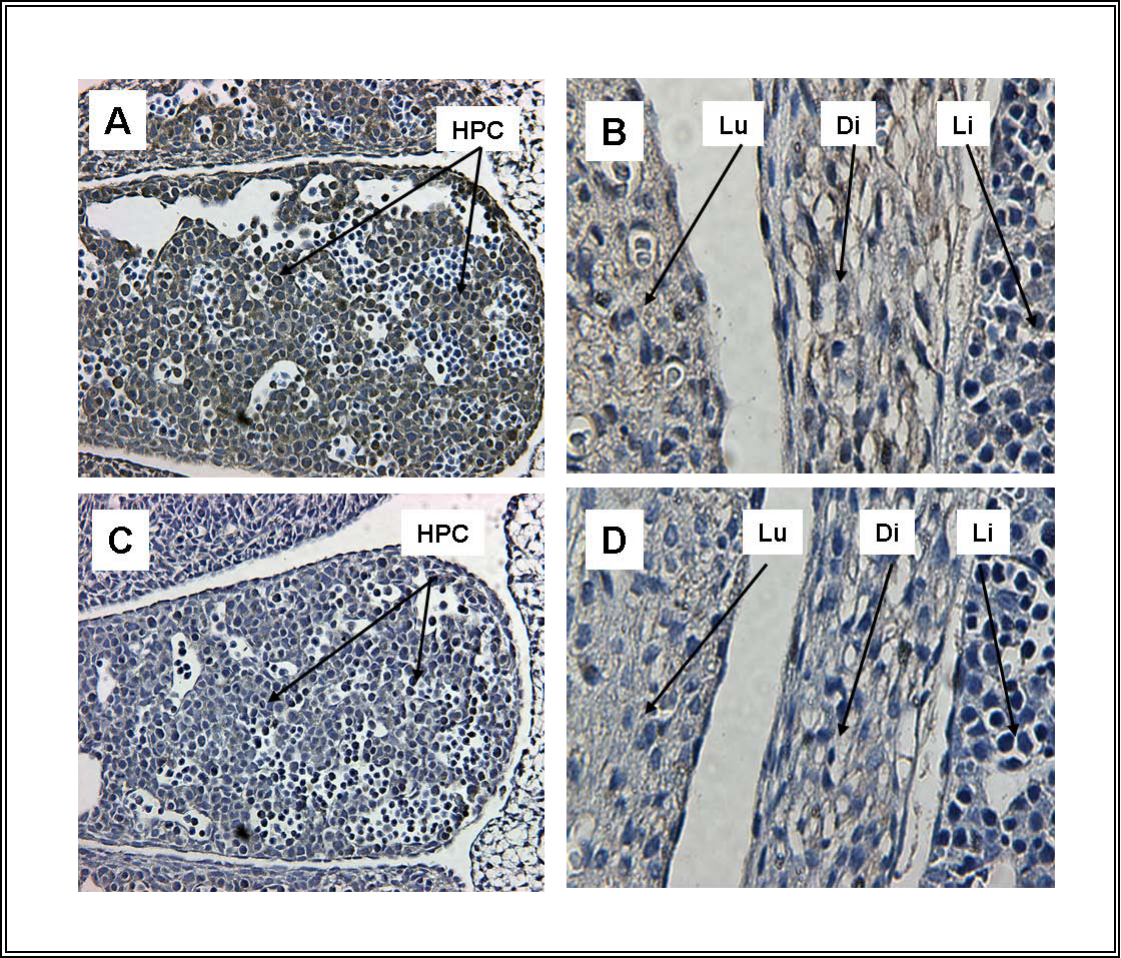
**Liver at E11 (A, C) and at E15 (B, D)**. A. TFII-I is present in a principally cytoplasmic distribution in liver. B. Sections through lung (Lu), diaphragm (Di) and liver (Li) demonstrate differential levels of TFII-I expression, with higher levels in lung and diaphragm than in liver. C and D. Immunoreactivity is competed completely by pre-incubation of the antibody with peptide. Abbr.: Di - Diaphragm, HPC-Hepatic Primordial Cells, Li - Liver, Lu - Lungs,

Sections through liver (Figure 3A) show widespread, low-level expression of TFII-I in developing hepatocytes. This immunoreactivity is fully blocked by pre-incubation of the antibody with peptide as described in methods (Figure 3C, D).

**Figure 4:**
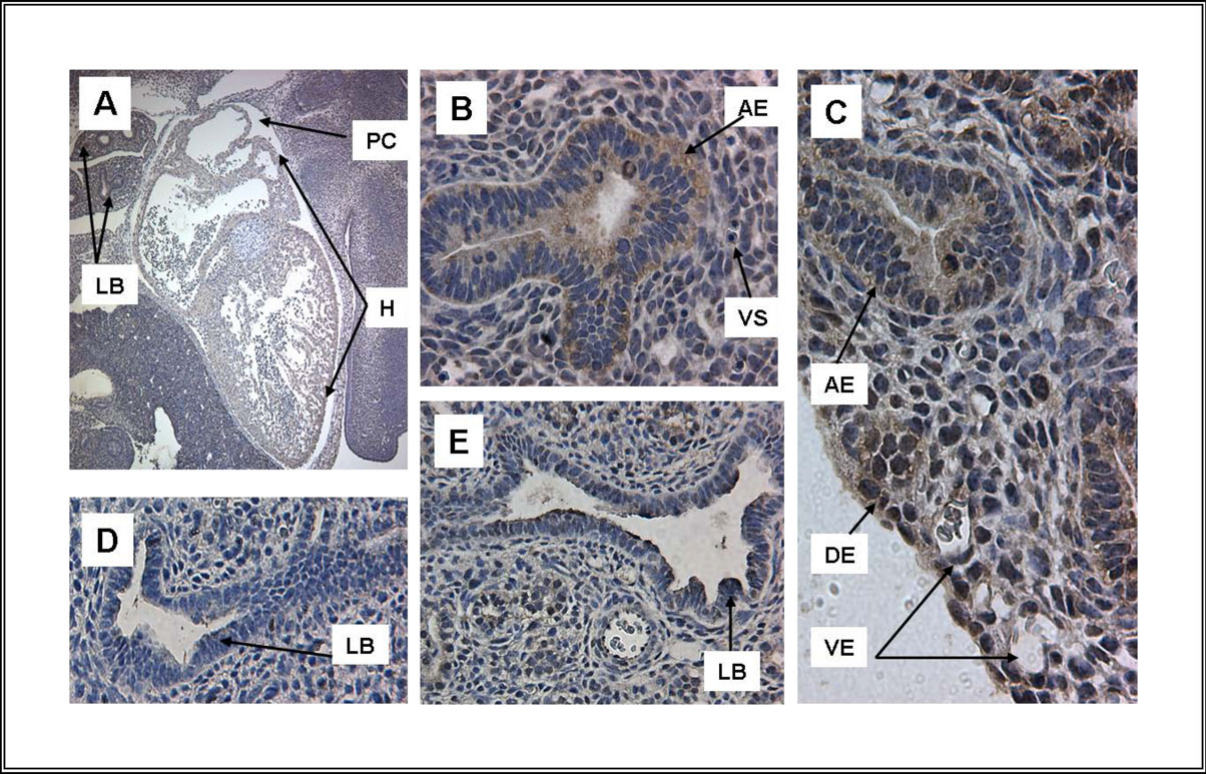
**Heart and developing lung demonstrate TFII-I expression**. A. Heart at E12 (5 ×), B. Lung at E12, C. Lung at E16, D and E. Lung at E15. A low power view of the ED12 embryo shows extensive, largely cytoplasmic TFII-I immunoreactivity in heart. Both E12 and E16 are representative of the pattern of TFII-I expression in lung (64 ×), showing extensive immunoreactivity in developing airway epithelium as well as in endothelial cells lining the vasculature (arrows). Immunoreactivity is present in lung parenchyma with increased intensity and nuclear localization at E16 compared with E12. Immuno-reactivity detected in lung at E15 (Figure 4 E) is competed completely by pre-incubation of the antibody with peptide (Figure 4D). Abbr.: AE-Airway Epithelium, DE- Developing Epithelium, H-Heart, LB-Lobar Bronchus, PC-Pericardial Cavity, VE-Vascular Endothelium, VS-Vascular System,

The expanding lung buds at E12 (Figure 4B) express high levels of TFII-I in the airways, vasculature and parenchyma. This pattern is demonstrated throughout embryonic development (Figure 4E, C) and is discussed in more detail below. In addition to expression in the pulmonary system, TFII-I is highly expressed in developing heart (Figure 4A). Expression in the brain is also noted (Figure 2E) and contrasts with that of the related transcription factor, TFII-IRD1 (Figure 2F), as we have previously described. TFII-I immunoreactivity is also noted in the developing nasopharynx (Figure 2G).

**Figure 5:**
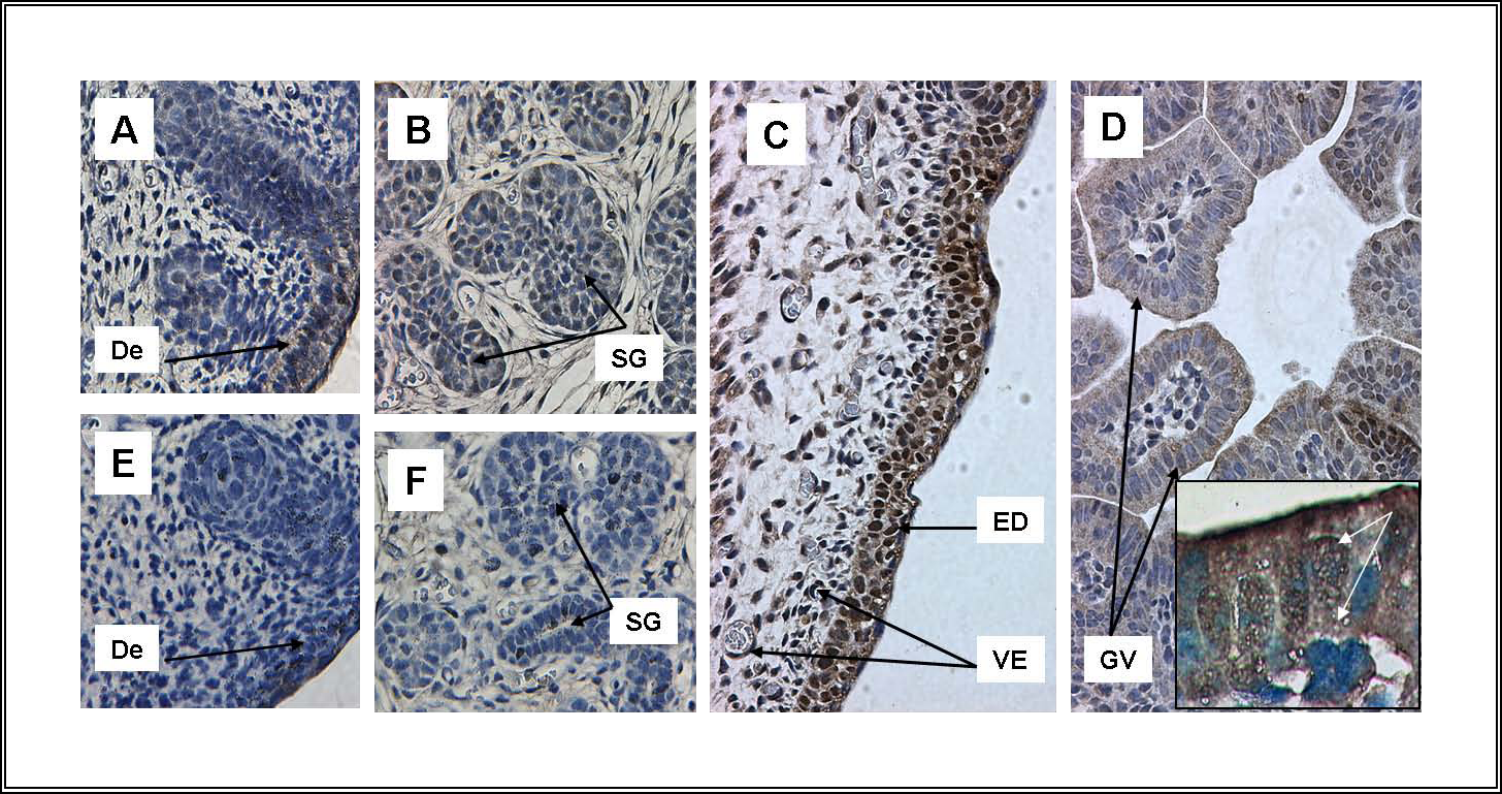
**Expression of TFII-I in multiple tissues at E15 and E16**. A. Skin and hair follicles, B. Submandibular gland, C. Dermis (E16), D. Gut Vill (E16), E-F. Skin and hair follicles and submandibular gland lacking immunoreactivity after pre-incubation of antibody with peptide. TFII-I expression is present in multiple tissues at ED15 and the immunoreactivity is completely competed by cognate peptide. TFII-I is expressed in skin and hair follicles (A) and in submandibular glands (B). Dermis demonstrates extensive immunoreactivity both in the superficial dermal layers and in underlying mesenchyme and vessels. TFII-I immunoreactivity is prominent in nucleus as well as cytoplasm in the dermis. D. Gut villi show both cytoplasmic and nuclear immunoreactivity particularly at the apical surface. Again, immunoreactivity is competed completely by pre-incubation of the antibody with peptide (E-F). Inset in Figure 5D shows nuclear and cytoplasmic locatization of TFII-I under 100 × magnification (Zeiss Axioscop, obj. Apochromat 100 ×). White arrows indicate positive reaction of specific antibody with TFII-I. Abbr.: De-Dermis, ED - Epidermis, GV-Gut Villi, SG-Submandibular Gland, VE - Vascular Endothelium

A survey of tissues from E15 demonstrates expression of TFII-I in multiple organ systems (Figures 3B, 4E, C, 5A-D). High levels of TFII-I expression are noted in skin and hair follicles (Figure 5A) as well as in submandibular glands (Figure 5B). Lung continues to show a similar pattern of expression to earlier time points (Figure 4E, C).

The relative expression of TFII-I in lung, diaphragm and liver is highlighted in Figure 3B with lung and diaphragm showing more abundant expression than liver. Again, immunoreactivity is eliminated by pre-incubation of the antibody with peptide (Figure 3C, D, 4D).

Similar patterns of expression are also seen at E16. Intense immunoreactivity is noted in skin (Figure 5C) as well as in subcutaneous vasculature (Figure 5A, B). Gut also demonstrates TFII-I expression, but at a lower level (Figure 5D). Lung continues to express TFII-I in the pattern seen at earlier stages (Figure 4C).

### Expression of TFII-I by Organ Systems

As described above by developmental stage, TFII-I expression is widespread in developing mouse embryo. This, however, is not uniform, but instead shows variation in amount as well as sub-cellular distribution. Further, the expression appears to follow specific patterns based on organ system. These patterns are summarized in the sections below.

### Expression in Vasculature, Heart and Lung

TFII-I protein has been detected in adult vasculature, endothelial cells, heart and lung. We examined the timing and localization of TFII-I in developing vascular structures and in heart as well as in lung. Expression was detected in vasculature beginning at E11 (data not shown). We were unable to identify vascular structures at earlier time points. In later time-points (Figure 4, 5), TFII-I immunoreactivity was noted in vascular endothelial cells in lung and dermis as well as in adjacent smooth muscle cells.

Heart showed high levels of TFII-I expression beginning at E11 (not shown) and throughout development (Figure 4A). Expression was uniform throughout the heart.

TFII-I immunoreactivity was noted throughout lung development. Staining was most intense in the airway epithelium early in development (Figure 4B). Later sections show staining in the adjacent vasculature as well as in the developing air sacs (Figure 4E, C). This pattern is also detected in adult mouse lung (not shown). The nasopharynx, the external extreme of the respiratory system, also expresses TFII-I from E12 (Figure 2G).

### Expression of TFII-I in Gastrointestinal Tract and Liver

TFII-I expression is detected in the developing gut. Expression is most intense in the villi of the gut (Figure 5D). The liver also shows diffuse, low-level immunoreactivity as early as E11 (Figure 3A). This pattern is also noted at E15 (Figure 3B).

### Expression of TFII-I in Glands and Skin

Significant TFII-I staining is noted in the skin and glands. Beginning at E11, skin and developing hair follicles show high levels of expression similar to those seen at E15 (Figure 5A). Significant TFII-I expression is also detected in the submandibular glands as noted at E15 (Figure 5B).

### Expression of TFII-I in Central Nervous System

The presence of TFII-I has been examined both in development and adult brain because of its putative association with Williams-Beuren Syndrome [7]. We find that TFII-I is expressed in a regionally defined fashion within the CNS consistent with previous reports (Figure 1 and 2). Further, we find that TFII-I and TFII-IRD1 demonstrate distinct patterns of expression in embryo (Figure 2E and 2F) as observed in adult mouse.

## Discussion

In early development only trace or low levels of *Gtf2i *were detected between 1-cell stage and E3.5. At day E3.5 strong signal in inner cell mass and moderate signal in trophoectoderm of blastocyst were revealed by direct immuno-fluorescence, RT-PCR and WISH (Whole Mount in Situ RNA Hybridization). For more details see Additional File 3.

Our studies have identified TFII-I protein expression in post-implantation embryos since the beginning of organogenesis, from E8 until E16. We found that in developmental period spanned by our study TFII-I was continuously present in the embryo, although it's spatial and temporal distribution varied, as well as its sub-cellular localization (see Table 1).

**Table 1:** TFII-I expression during organogenesis, as detected by IHC. Developing organs and tissues were identified using EMAGE gene expression database [12] and [25]

DEVELOPMENTAL STAGE	ORGAN	LOCALIZATION
ED8.0 Embryo, Trophoectodermal derivatives	Neural ectoderm, mesoderm	Cytoplasmic (Fig. 1)

ED9.0 Embryo:	Neural tube (brain region) Inner and outer retinal layer Eye primordium	Nuclear, cytoplasmic (Fig. 2)

ED11 Embryo	Liver: developing hepatocytes Vasculature: lining endothelial cells Nasopharynx	Nuclear, cytoplasmic (Fig. 3A)

ED12 Embryo	Brain Nasopharynx Heart Lung buds: developing airway epithelium, endothelial cells	Nuclear, cytoplasmic (Fig. 2E) Cytoplasmic (Fig. 2G) Cytoplasmic (Fig. 4A) Nuclear, cytoplasmic (Fig. 4B, E, 4C)

ED15 Embryo	Skin and hair follicles Submandibular gland Lung: epithelium, endothelial cells	Cytoplasmic (Fig. 5A) Nuclear (Fig. 5B) Nuclear, cytoplasmic (Fig. 4E)

ED16 Embryo	Dermis: superficial dermal layers, underlying mesenchyme, blood vessels Gut: villi Lung: airway epithelium, vascular endothelial cells, developing alveolar epithelium	Nuclear, cytoplasmic (Fig. 5A) Nuclear, cytoplasmic (Fig. 5D) Nuclear (Fig. 4C)

In adult animal TFII-I regulates expression of VEGFR-2 (Flk1, Kdr), a receptor critical in differentiation and angiogenesis [13,14]. In mouse development Kdr can be detected as early as in E6.25 in embryo [15] and in E8.5 in extra-embryonic regions, in parallel to formation of blood islands [16]. The fact that both genes are expressed at the same time may suggest that TFII-I control over Kdr launches early in embryonic life. It was shown recently that homozygous deletion of *Gtf2i *caused embryonic lethality due to defects in yolk sac vasculogenesis and angiogenesis: *Gtf2i *inactivation resulted in downregulation of the VEGFR2, which deteriorated vascular signaling [6].

We found high levels of TFII-I in the airway epithelium, starting at E11. This finding complements other study which showed expression of TFII-I in the epithelium and in the underlying mesenchyme during odontogenesis in mouse embryo, between day E12.5 and newborn [17].

Our data showing the presence of TFII-I in the embryo confirm its role in cell differentiation and organogenesis, although a mechanism of its interactions is not clear. In cultured cells TFII-I is capable of activating the cyclin D1 gene and thus inducing transition from phase G1 into phase S of the cell cycle [18]. Cell entry into phase S, phase S progression and entry into phase G2/M were significantly delayed in TFII-I knocked down cells. It is postulated that in early phases of cell cycle TFII-I may also control PKC-β which additionally activates cyclin D1 via NFκB pathway. TFII-I itself can be phosphorylated by cyclin dependent kinase 1 (Cdk1) thus amplifying phase transition signals [19]. First cyclins expressed in mouse embryo are cyclin b1 and b2, detected in the inner cell mass and the trophectoderm of the blastocyst at E4.5 [20], followed by cyclins D and E at E7.5 [21]. Again, whether such an interaction between TFII-I and cyclins occurs at this early development stage, remains to be revealed.

## Conclusions

Based on our observations and others [2,6,8,17,22-24] TFII-I is critical in early mouse development. The structure makes it uniquely capable of transducing multiple regulatory signals as occurs in development. Our data add to the existing knowledge on TFII-I function as a pleiotropic transcription factor.

## List of abbreviations used

Ab: antibody; BR: basic region; cdk1: cyclin dependent kinase 1; EST: expressed sequence tag; GTF2-I: General Transcription Factor II-I; HLH: helix-loop-helix; Inr: initiator; MMP-9: matrix metalloproteinase 9: NGS: normal goat serum; PLCγ: phospholipase C gamma; TFII-IRD1: Transcription Factor that shares similarity with TFII-I in the 95-amino-acid HLH-like I-repeat domain (other names: BEN/WBSCR11/MusTRD1/Cream1); TRPC3: transient receptor potential calcium channel; VEGFR2: Vascular endothelial growth factor receptor 2; WBS: Williams-Beuren Syndrome; WISH: whole mount in situ hybridization

## Competing interests

The authors declare that they have no competing interests.

## Authors' contributions

IF analyzed immunohistochemistry and revised the manuscript. DS performed immunohistochemistry and assisted in the drafting of the manuscript. CB provided critical revisions to the manuscript. SD oversaw the project as a whole and drafted the manuscript.

## Supplementary Material

Additional file 1 **Structure, Functions and Cellular Localization of TFII-I**. Click here for file

Additional file 2 **TFII-I Target Genes**. Click here for file

Additional file 3 **Developmental Expression of TFII-I mRNA and Reported Expression of TFII-I in Mouse Development**. Click here for file

Additional file 4 **Methods**. Click here for file
