# Supplementary material for: Expression of the transcription factor, TFII-I, during post-implantation mouse embryonic development
Source: BMC Res Notes. 2010 Jul 20;3:203. doi: 10.1186/1756-0500-3-203 (PMC2921380; doi:10.1186/1756-0500-3-203)
Supplement: Additional file 1 — Structure, Functions and Cellular Localization of TFII-I. [file 1756-0500-3-203-S1.DOC]

***Expression of the transcription factor, TFII-I, during post-implantation mouse embryonic development****,* Fijalkowska I, Sharma D, Bult JC, Danoff SK

**Additional File 1: Structure, Functions and Cellular Localization of TFII-I**

Structurally, the TFII-I protein comprises several domains that define its biological function: an N-terminal leucine zipper domain is followed by six I-repeats (R1-R6), each containing a helix-loop-helix (HLH) domain. Leucine zipper is involved in dimerization, whereas sequence between the first and second repeat includes a basic DNA binding domain (BR), nuclear localization signal, two Src auto phosphorylation sites, MAPK interaction site, polyproline II domain and SH3 binding domain [1]. The helix-loop-helix motif within each I-repeat (R1-R6), by analogy to other transcription factors of similar structure, may be responsible for DNA binding or protein–protein interactions [2-4]. All repeats have similar folds, however, each domain is unique in terms of surface electrostatic charge distribution [5], which contributes to functional variation of TFII-I isoforms. Figure 6 shows schematic diagram of TFII-I protein structure.

Four isoforms of TFII-I, which are generated by alternative mRNA splicing are known to differ from each other in their expression patterns in various tissues and among species [6-8]. For instance, two spliced isoforms of TFII-I were found in murine fibroblasts: TFII-I delta and beta, which are of distinct subcellular localization and antagonistic transcription functions [9]. Overlapping functions between short truncated isoforms may suggest certain self-regulation mode of action for each TFII-I [10]. Overall, TFII-I has the potential of differentially regulating its target genes via homo- or heteromerization of its isoforms. These, in turn, may have distinct functions at both the cellular and species levels. The complexity of TFII-I interplay is further amplified through its interaction with other helix-loop-helix transcription factors [2, 11].

The related transcription factor, TFII-IRD1, shares the non-canonical HLH domains with TFII-I [12]. TFII-IRD1 has been shown to antagonize TFII-I at a number of promoters [13, 14]. The locus for TFII-IRD1 is adjacent to that of TFII-I, which has contributed to the speculation that TFII-IRD1 arose as a result of gene duplication from TFII-I [12]. Similarly to *Gtf2i*, homozygous loss of *Gtf2ird1* results in embryonic lethality [15].

TFII-I protein resides in the cytoplasm in a basally phosphorylated form at serine/threonine and tyrosine residues [16]. Upon c-Src-dependent tyrosine phosphorylation on residues 248 and 611, TFII-I translocates to the nucleus, where it reversibly activates a c-fos gene [17]. Similar induction occurs *via* Btk-dependent phosphorylation of TFII-I on Tyr248, Tyr357, and Tyr462 sites [18]. However, Src-dependent phosphorylation does not compete with Btk mediated phosphorylation, which implies that multiple kinases can independently target TFII-I. Upon phosphorylation and translocation to the nucleus TFII-I activates transcription machinery by interacting with both basal initiator (Inr) and upstream regulatory sites (E box) in multiple genes, as well as with USF1 and c-*myc* [19-21]. Thus, TFII-I links extracellular events to nuclear gene expression.

Beyond its transcriptional function in the nucleus, cytoplasmic TFII-I negatively regulates agonist-induced calcium entry by competitive binding to phospholipase C-γ (PLC-gamma) and blocking surface transient receptor potential calcium channels (TRPC3) [22]. Since alterations in calcium levels affect a wide range of signaling pathways, TFII-I may act as a potent modulator of cellular events both at a transcription and non-transcription level [23].

**Figure 6. Schematic diagram of TFII-I protein structure, isoform 1.** Six R (I-like) domains are represented by a picture of the structural model of the domain. The R domains within TFII-I molecule cover amino acid sequences as follows: R1:103-197, R2:352-446, R3:457-551, R4:562-656, R5:724-818, R6:859-953. Leucin zipper (LZ) domain spans aa2-90; nuclear localization site (NLS) covers aa320-327; and poly Asn site (pAsn) is located within aa692-695. Candles indicate two Src phosphorylation sites within aa244-248 and aa273-277. BR represents putative DNA binding sites. Other binding sites localized within the region between R1 and R2 are as follows: M represents binding site (aa282-293) for MAPK, S represents SH3 binding motive, P represents polyproline II domain. Portions of the sequence labeled as A (aa 255-274) and B (aa294-314) are products of exon A and B, respectively, and are missing in isoforms. Isoform 1 (identifier: P78347-1) is a full length protein of 998 aa; isoform 2 (identifier: P78347-2) contains 957aa with missing blocks A and B; isoform 3 (identifier: P78347-3) contains 978 aa with missing block A; isoform 4 (identifier: P78347-4) contain 977 aa and no block B. Information on isoform sequences was from (UniProtKB/Swiss-Prot P78347 (GTF2I_HUMAN) database and from [1]. TFII-I Isoform 1 sequence (P78347-1) was used as a canonical and all annotations are referred accordingly. The picture of I-like domain structure used to create the diagram was copied from RCSP Protein Data Bank (RCSB PDB), viewed with Ligand Explorer 3.5 (powered by the MBT) and modified with Microsoft Office Picture Manager to maintain N-terminal to C-terminal direction of amino acid sequence within the protein structure. Website for PBD: <http://www.rcsb.org/pdb/explore/literature.do?structureId=2ED2>,

**References**

1. AL Roy: **Biochemistry and biology of the inducible multifunctional transcription factor TFII-I**. *Gene* 2001, **274**:1-13.

2. LA Perez Jurado, YK Wang, R Peoples, A Coloma, J Cruces, U Francke: **A duplicated gene in the breakpoint regions of the 7q11.23 Williams-Beuren syndrome deletion encodes the initiator binding protein TFII-I and BAP-135, a phosphorylation target of BTK**. *Hum Mol Genet* 1998, **7**:325-34.

3. AK Ewart, CA Morris, D Atkinson, W Jin, K Sternes, P Spallone, AD Stock, M Leppert, MT Keating: **Hemizygosity at the elastin locus in a developmental disorder, Williams syndrome**. *Nat Genet* 1993, **5**:11-16.

4. LR Osborne, M Li, B Pober, D Chitayat, J Bodurtha, A Mandel, T Costa, T Grebe, S Cox, LC Tsui, et al: **A 1.5 million-base pair inversion polymorphism in families with Williams-Beuren syndrome**. *Nat Genet* 2001, **29**:321-5.

5. U DeSilva, L Elnitski, JR Idol, JL Doyle, W Gan, JW Thomas, S Schwartz, NL Dietrich, SM Beckstrom-Sternberg, JC McDowell, et al: **Generation and comparative analysis of approximately 3.3 Mb of mouse genomic sequence orthologous to the region of human chromosome 7q11.23 implicated in Williams syndrome**. *Genome Res* 2002, **12**:3-15.

6. U Bellugi, L Lichtenberger, D Mills, A Galaburda, JR Korenberg: **Bridging cognition, the brain and molecular genetics: evidence from Williams syndrome**. *Trends Neurosci* 1999, **22**:197-207.

7. AM Galaburda, D Holinger, D Mills, A Reiss, JR Korenberg, U Bellugi: **[Williams syndrome. A summary of cognitive, electrophysiological, anatomofunctional, microanatomical and genetic findings]**. *Rev Neurol* 2003, **36 Suppl 1**:S132-7.

8. A Karmiloff-Smith, J Grant, S Ewing, MJ Carette, K Metcalfe, D Donnai, AP Read, M Tassabehji: **Using case study comparisons to explore genotype-phenotype correlations in Williams-Beuren syndrome**. *J Med Genet* 2003, **40**:136-40.

9. B Enkhmandakh, AV Makeyev, L Erdenechimeg, FH Ruddle, NO Chimge, MI Tussie-Luna, AL Roy, D Bayarsaihan: **Essential functions of the Williams-Beuren syndrome-associated TFII-I genes in embryonic development**. *Proc Natl Acad Sci U S A* 2009, **106**:181-6.

10. AL Roy, H Du, PD Gregor, CD Novina, E Martinez, RG Roeder: **Cloning of an inr- and E-box-binding protein, TFII-I, that interacts physically and functionally with USF1**. *EMBO J* 1997, **16**:7091-104.

11. ME Massari, C Murre: **Helix-Loop-Helix Proteins: Regulators of Transcription in Eucaryotic Organisms**. *Mol. Cell. Biol.* 2000, **20**:429-440.

12. D Vullhorst, A Buonanno: **Multiple GTF2I-like repeats of general transcription factor 3 exhibit DNA binding properties. Evidence for a common origin as a sequence-specific DNA interaction module**. *J Biol Chem* 2005, **280**:31722-31.

13. Y Doi-Katayama, F Hayashi, M Inoue, T Yabuki, M Aoki, E Seki, T Matsuda, T Kigawa, M Yoshida, M Shirouzu, et al: **Solution structure of the general transcription factor 2I domain in mouse TFII-I protein**. *Protein Sci* 2007, **16**:1788-1792.

14. V Cheriyath, AL Roy: **Alternatively spliced isoforms of TFII-I. Complex formation, nuclear translocation, and differential gene regulation**. *J Biol Chem* 2000, **275**:26300-8.

15. SK Danoff, HE Taylor, S Blackshaw, S Desiderio: **TFII-I, a candidate gene for Williams syndrome cognitive profile: parallels between regional expression in mouse brain and human phenotype**. *Neuroscience* 2004, **123**:931-8.

16. AV Makeyev, D Bayarsaihan: **Alternative splicing and promoter use in TFII-I genes**. *Gene* 2009, **433**:16-25.

17. S Hakre, MI Tussie-Luna, T Ashworth, CD Novina, J Settleman, PA Sharp, AL Roy: **Opposing functions of TFII-I spliced isoforms in growth factor-induced gene expression**. *Mol Cell* 2006, **24**:301-8.

18. TA Hinsley, P Cunliffe, HJ Tipney, A Brass, M Tassabehji: **Comparison of TFII-I gene family members deleted in Williams-Beuren syndrome**. *Protein Sci* 2004, **13**:2588-99.

19. AL Roy, C Carruthers, T Gutjahr, RG Roeder: **Direct role for Myc in transcription initiation mediated by interactions with TFII-I**. *Nature* 1993, **365**:359-61.

20. Y Franke, RJ Peoples, U Francke: **Identification of GTF2IRD1, a putative transcription factor within the Williams-Beuren syndrome deletion at 7q11.23**. *Cytogenet Cell Genet* 1999, **86**:296-304.

21. TA Jackson, HE Taylor, D Sharma, S Desiderio, SK Danoff: **Vascular endothelial growth factor receptor-2: counter-regulation by the transcription factors, TFII-I and TFII-IRD1**. *J Biol Chem* 2005, **280**:29856-63.

22. D Tantin, MI Tussie-Luna, AL Roy, PA Sharp: **Regulation of immunoglobulin promoter activity by TFII-I class transcription factors**. *J Biol Chem* 2004, **279**:5460-9.

23. CD Novina, V Cheriyath, AL Roy: **Regulation of TFII-I activity by phosphorylation**. *J Biol Chem* 1998, **273**:33443-8.
