# Supplementary material for: Expression of the transcription factor, TFII-I, during post-implantation mouse embryonic development
Source: BMC Res Notes. 2010 Jul 20;3:203. doi: 10.1186/1756-0500-3-203 (PMC2921380; doi:10.1186/1756-0500-3-203)
Supplement: Additional file 2 — TFII-I Target Genes. [file 1756-0500-3-203-S2.DOC]

***Expression of the transcription factor, TFII-I, during post-implantation mouse embryonic development****,* Fijalkowska I, Sharma D, Bult JC, Danoff SK

**Additional File 2: TFII-I Target Genes**

So far, direct involvement of TFII-I in gene regulation has been confirmed for more than 20 genes. Table 2 presents a list of genes that contain TFII-I binding sites in their sequences and were found controlled by TFII-I, by methods other than gene arrays. The list covers target genes studied in humans and in other species, as indicated. Some of genes were found up-, some down-regulated, others were only reported to interact with the transcription factor. Table 2 presents an overview of these findings.

**Table 2.** **TFII-I-Responsive Promoters and Associated Gene Function.** Entrez Gene ids of promoters affected by TFII-I are indicated. Arrows indicate ↑ up-, or ↓down regulation of a target gene. Both ↑↓ arrows indicate that TFII-I may act either as enhancer or as a suppressor, depending on the context of other factors. Lack of arrow indicates that the TFII-I binding sequence was found within the gene but its effect was not defined.

| PROMOTER REGULATED BY TFII-I | FUNCTION OF GENE(S) EXPRESSED | REFERENCE |
| --- | --- | --- |
| ARAF1, *Homo sapiens*, GeneID: 369 | Proto-oncogene with cytoplasmic protein kinase activity. Plays a central role in cell growth and development. | [1] |
| ↓ Beta-globin, *Homo sapiens,*  GeneID: 64162 | Required for expression of beta polypeptide chains in adult hemoglobin. | [2-4] |
| ↑ CD3delta (mCD3d), *Mus musculus,* GeneID: 12500 | Regulates commitment to the development of the T cell lineage | [5] |
| ↑ c-fos, *Mus musculus,* GeneID: 14281 | Regulates cellular proliferation, differentiation, transformation, and apoptosis. | [6, 7] |
| ↓ Cyclin D1 GeneID: 595, Cyclin D2 GeneID: 894, Cyclin D3 GeneID: 896, *Homo sapiens* | Regulate cell cycle progression from G1 to S phase, cellular proliferation and cdk activity. | [8, 9] |
| ↑↓ Downstream Immunoglobulin Control Element (DICE) | Cis element downstream of the transcription initiation site of murine heavy chain variable promoters, critical for B cell activity and specificity. | [10] |
| ↑↓ DYX1C1, *Homo sapiens*, GeneID: 161582 | Candidate gene for dyslexia susceptibility. Plays role in controlling neuronal migration during embryogenesis. Has an effect on learning in rodents. | [11] |
| E2F2, *Homo sapiens*, GeneID:1870 | Plays a role in cell cycle regulation, proliferation and tumor suppressor activity. | [9] |
| ↓ Estrogen Responsive genes*, Homo sapiens,* pS2 GeneID: 7031,  amphiregulin GeneID: 374 | Estrogen receptors (nuclear) function as ligand-dependent transcriptional regulators of estrogen-responsive genes. | [3, 12] |
| ↑ Glucose Regulated Proteins containing Endoplasmic Reticulum Stress Response Elements (ERSE): Erp72 (*Mus musculus*, GeneID: 12304 | Endoplasmic reticulum chaperones. | [13] |
| ↑ Goosecoid (Gsc) *Xenopus tropicalis,* GeneID: 549458 | Regulates formation and patterning of embryos in response to TGF beta/activin stimulation. | [14] |
| ↑ Grp78, *Homo sapiens*, GeneID:3309 | Involved in the folding and assembly of proteins in the ER. Regulates activation of ER stress signal transducers. | [13, 15] |
| L1 – Neural Cell Adhesion Molecule, *Homo sapiens*, GeneID: 3897 | Mediates cell interactions in the developing and regenerating nervous system of mammals | [16] |
| ↑ Luteinizing Hormone Receptor (rLHR), *Rattus norvegicus,* GeneID: 25477 | Mediates cellular response to luteinizing hormone and choriogonadotropin. | [17] |
| Mullerian Inhibiting Substance (MIS), *Homo sapiens*, GeneID: 268 | Regulates mammalian male sexual development. | [18] |
| Ribonucleotide reductase R1, *Mus musculus*, GeneID: 20135 | Catalyzes reduction of ribonucleotides to deoxyribonucleotides. | [19] |
| ↑ Rous Sarcoma Virus Long Terminal Repeat (RSV LTR) | Promotes activity of adjacent cellular oncogenes. Involved in viral DNA synthesis. | [20] |
| Selenocysteine tRNA gene transcription-activating factor (mStaf), *Mus musculus*, GeneID: 20841 | Enhances transcription of the tRNASec gene. Stimulates biosynthesis of selenoproteins involved in lactation. | [21] |
| Thromboxane A2 synthase (TXAS), Homo sapiens, GeneID: 6916 | Catalyzes the biosynthesis of thromboxane A2 (TXA), a potent vasoconstrictor and mediator of platelet aggregation. | [22] |
| Vascular endothelial growth factor receptor 2 (VEGFR2), *Homo sapiens*, GeneID: 3791 | Regulates cellular response to VEGF. Implicated in vascular development and angiogenesis. | [23, 24] |
| V-Beta, T-cell receptor, Mus musculus, | Involved in V(D)J recombination in T cells and expression of the T cell receptor beta chain. | [25] |
| ↑ 5' long terminal repeats (LTRs) of HIV | Involved in reactivation of HIV-1 replication from latency in response to T-cell receptor engagement and RAS-MAPK activation. | [26-28] |

Recently, transcriptional potency of TFII-I was tested by a microarray: the protein was over-expressed in primary mouse embryonic fibroblasts and resulting alterations in gene expression were monitored by a microarray. Among as many as 1,235 TFII-I- affected genes were those involved in the immunity response, catalytic activity, signaling pathways and transcriptional regulation [29]. On the other hand, targeted disruption of *Gtf2i* and *Gtf2ird1* in mouse embryonic stem cells revealed significant down-regulation of three so far unknown genes: Scand3, 4933436H12Rik and Kbtbd7 that contain TFII-I binding sequences in their promoters and may be direct targets of TFII-I. It was concluded that impaired expression of these proteins, due to chromosomal deletions in Williams-Beuren syndrome and resulting TFII-I insufficiency, may contribute to the etiology of the disease [30].

References

1. J-E Lee, TW Beck, U Brennscheidt, LJ deGennaro, UR Rapp: **The Complete Sequence and Promoter Activity of the Human A-raf-1 Gene (ARAF1)**. *Genomics* 1994, **20**:43-55.

2. KM Leach, KF Vieira, SH Kang, A Aslanian, M Teichmann, RG Roeder, J Bungert: **Characterization of the human beta-globin downstream promoter region**. *Nucleic Acids Res* 2003, **31**:1292-301.

3. VJ Crusselle-Davis, KF Vieira, Z Zhou, A Anantharaman, J Bungert: **Antagonistic regulation of beta-globin gene expression by helix-loop-helix proteins USF and TFII-I**. *Mol Cell Biol* 2006, **26**:6832-43.

4. VJ Crusselle-Davis, Z Zhou, A Anantharaman, B Moghimi, T Dodev, S Huang, J Bungert: **Recruitment of coregulator complexes to the beta-globin gene locus by TFII-I and upstream stimulatory factor**. *FEBS J* 2007, **274**:6065-73.

5. HB Ji, A Gupta, S Okamoto, MD Blum, L Tan, MB Goldring, E Lacy, AL Roy, C Terhorst: **T cell-specific expression of the murine CD3delta promoter**. *J Biol Chem* 2002, **277**:47898-906.

6. DA Grueneberg, RW Henry, A Brauer, CD Novina, V Cheriyath, AL Roy, M Gilman: **A multifunctional DNA-binding protein that promotes the formation of serum response factor/homeodomain complexes: identity to TFII-I**. *Genes Dev* 1997, **11**:2482-93.

7. DW Kim, V Cheriyath, AL Roy, BH Cochran: **TFII-I enhances activation of the c-fos promoter through interactions with upstream elements**. *Mol Cell Biol* 1998, **18**:3310-20.

8. ZP Desgranges, J Ahn, MB Lazebnik, T Ashworth, C Lee, RC Pestell, N Rosenberg, C Prives, AL Roy: **Inhibition of TFII-I-dependent cell cycle regulation by p53**. *Mol Cell Biol* 2005, **25**:10940-52.

9. T Stasyk, A Dubrovska, M Lomnytska, I Yakymovych, C Wernstedt, CH Heldin, U Hellman, S Souchelnytskyi: **Phosphoproteome profiling of transforming growth factor (TGF)-beta signaling: abrogation of TGFbeta1-dependent phosphorylation of transcription factor-II-I (TFII-I) enhances cooperation of TFII-I and Smad3 in transcription**. *Mol Biol Cell* 2005, **16**:4765-80.

10. D Tantin, MI Tussie-Luna, AL Roy, PA Sharp: **Regulation of immunoglobulin promoter activity by TFII-I class transcription factors**. *J Biol Chem* 2004, **279**:5460-9.

11. I Tapia-Paez, K Tammimies, S Massinen, AL Roy, J Kere: **The complex of TFII-I, PARP1, and SFPQ proteins regulates the DYX1C1 gene implicated in neuronal migration and dyslexia**. *FASEB J.* 2008, **22**:3001-3009.

12. Y Ogura, M Azuma, Y Tsuboi, Y Kabe, Y Yamaguchi, T Wada, H Watanabe, H Handa: **TFII-I down-regulates a subset of estrogen-responsive genes through its interaction with an initiator element and estrogen receptor alpha**. *Genes Cells* 2006, **11**:373-81.

13. R Parker, T Phan, P Baumeister, B Roy, V Cheriyath, AL Roy, AS Lee: **Identification of TFII-I as the endoplasmic reticulum stress response element binding factor ERSF: its autoregulation by stress and interaction with ATF6**. *Mol Cell Biol* 2001, **21**:3220-33.

14. M Ku, SY Sokol, J Wu, MI Tussie-Luna, AL Roy, A Hata: **Positive and negative regulation of the transforming growth factor beta/activin target gene goosecoid by the TFII-I family of transcription factors**. *Mol Cell Biol* 2005, **25**:7144-57.

15. M Hong, MY Lin, JM Huang, P Baumeister, S Hakre, AL Roy, AS Lee: **Transcriptional regulation of the Grp78 promoter by endoplasmic reticulum stress: role of TFII-I and its tyrosine phosphorylation**. *J Biol Chem* 2005, **280**:16821-8.

16. G Chalepakis, J Wijnholds, P Giese, M Schachner, P Gruss: **Characterization of Pax-6 and Hoxa-1 binding to the promoter region of the neural cell adhesion molecule L1**. *DNA Cell Biol* 1994, **13**:891-900.

17. H Youn, Y Koo, I Ji, TH Ji: **An upstream initiator-like element suppresses transcription of the rat luteinizing hormone receptor gene**. *Mol Endocrinol* 2005, **19**:1318-28.

18. N Morikawa, TR Clarke, CD Novina, K Watanabe, C Haqq, M Weiss, AL Roy, PK Donahoe: **Human Mullerian-inhibiting substance promoter contains a functional TFII-I-binding initiator**. *Biol Reprod* 2000, **63**:1075-83.

19. E Johansson, E Skogman, L Thelander: **The TATA-less promoter of mouse ribonucleotide reductase R1 gene contains a TFII-I binding initiator element essential for cell cycle-regulated transcription**. *J Biol Chem* 1995, **270**:30162-7.

20. CM Mobley, L Sealy: **The Rous sarcoma virus long terminal repeat promoter is regulated by TFII-I**. *J Virol* 2000, **74**:6511-9.

21. K Adachi, M Katsuyama, S Song, T Oka: **Genomic organization, chromosomal mapping and promoter analysis of the mouse selenocysteine tRNA gene transcription-activating factor (mStaf) gene**. *Biochem J* 2000, **346 Pt 1**:45-51.

22. R Tazawa, ED Green, K Ohashi, KK Wu, LH Wang: **Characterization of the complete genomic structure of human thromboxane synthase gene and functional analysis of its promoter**. *Arch Biochem Biophys* 1996, **334**:349-56.

23. Y Wu, C Patterson: **The human KDR/flk-1 gene contains a functional initiator element that is bound and transactivated by TFII-I**. *J Biol Chem* 1999, **274**:3207-14.

24. TA Jackson, HE Taylor, D Sharma, S Desiderio, SK Danoff: **Vascular endothelial growth factor receptor-2: counter-regulation by the transcription factors, TFII-I and TFII-IRD1**. *J Biol Chem* 2005, **280**:29856-63.

25. V Cheriyath, CD Novina, AL Roy: **TFII-I regulates Vbeta promoter activity through an initiator element**. *Mol Cell Biol* 1998, **18**:4444-54.

26. J Chen, T Malcolm, MC Estable, RG Roeder, I Sadowski: **TFII-I regulates induction of chromosomally integrated human immunodeficiency virus type 1 long terminal repeat in cooperation with USF**. *J Virol* 2005, **79**:4396-406.

27. I Sadowski, DA Mitchell: **TFII-I and USF (RBF-2) regulate Ras/MAPK-responsive HIV-1 transcription in T cells**. *Eur J Cancer* 2005, **41**:2528-36.

28. T Malcolm, J Kam, PS Pour, I Sadowski: **Specific interaction of TFII-I with an upstream element on the HIV-1 LTR regulates induction of latent provirus**. *FEBS Letters* 2008, **582**:3903-3908.

29. NO Chimge, AV Makeyev, FH Ruddle, D Bayarsaihan: **Identification of the TFII-I family target genes in the vertebrate genome**. *Proc Natl Acad Sci U S A* 2008, **105**:9006-10.

30. AV Makeyev, D Bayarsaihan: **New TFII-I family target genes involved in embryonic development**. *Biochemical and Biophysical Research Communications* 2009, **386**:554-558.
