# Supplementary material for: Expression of the transcription factor, TFII-I, during post-implantation mouse embryonic development
Source: BMC Res Notes. 2010 Jul 20;3:203. doi: 10.1186/1756-0500-3-203 (PMC2921380; doi:10.1186/1756-0500-3-203)
Supplement: Additional file 3 — Developmental Expression of TFII-I mRNA and Reported Expression of TFII-I in Mouse Development. [file 1756-0500-3-203-S3.DOC]

***Expression of the transcription factor, TFII-I, during post-implantation mouse embryonic development****,* Fijalkowska I, Sharma D, Bult C, Danoff SK

**Additional File 3: Developmental Expression of TFII-I mRNA and Reported Expression of TFII-I in Mouse Development**

In order to expand on the available description of timing and location of the expression of TFII-I documented in the literature [1-3] we reviewed a number of resources on gene expression profile. Recognizing that each resource has some limitations, we sought to gain an overview of the expression pattern of TFII-I and its relationship with the related factor, TFII-IRD1, in development. Profiles of expressed sequence tags (ESTs) available from NCBI [4] demonstrate TFII-I mRNA expression in pre-implantation cDNA libraries (Table 3a). In comparison with TFII-IRD1, TFII-I message appears more abundant (based on transcripts per million) and is detected at any earlier stage. TFII-I message is detected throughout development and adult life, but expression is maximal during embryonic development.

To further delineate early expression, we reviewed data from The Jackson Laboratory on Mouse Gene Expression Database [5, 6] and EMAGE—Edinburgh Mouse Atlas of Gene Expression [7], summarized in the Table 3b. In agreement with EST expression profile is the observation that trace or low expression levels were detected in an early stage until E3.5 [8]. Other authors, however, detected TFII-I by RT-PCR in egg [3]. Immunohistochemical studies demonstrate presence of TFII-I protein in trophectoderm and deciduas at E4.5 during the pre-implantation period. In situ hybridization studies focused primarily on the central nervous system, detailing a complex pattern of regional expression at E13.5 and post-natal day 0 [9]. In situ hybridization studies in E14 mouse embryo suggested ubiquitous RNA expression, but with regional variation in intensity [10].

**Table 3a**. Expression profiles of TFII-I and TFII-IRD1 by analysis of EST counts. Data extracted from EST Profile Viewer (NCBI) [4, 11]. Developmental stages are defined as noted. Numerical data represent the number of transcripts per million (TPM). These numbers reflect a low-end estimate for the presence of a given transcript at a particular developmental stage, but suggest that TFII-I and TFII-IRD1 are expressed earlier than VEGFR-2.

| DEVELOPMENTAL STAGE | TFII-I  (Mm.261570) | TFII-IRD1  (Mm.332735) |
| --- | --- | --- |
| Egg | 0 | 153 |
| Pre-implantation | 202 | 63 |
| Post-implantation | 255 | 34 |
| Mid-gestation | 342 | 69 |
| Late-gestation | 468 | 89 |
| Neonate | 168 | 102 |
| Post natal | 150 | 27 |
| Adult | 152 | 34 |

**Table 3b**. Reported Expression of TFII-I in Mouse Development.

| DEVELOPMENTAL STAGE | STATUS | METHOD OF  DETECTION | REFERENCE |
| --- | --- | --- | --- |
| Unfertilized egg;    Zygote: pronuclei;  2-cell stage: nuclei, cytoplasm; polar body  nucleus;  8-16-cell and morula: inner cell mass (ICM),  trophectoderm;  ED3.5, early blastocyst: ICM, trophoecto-  derm  E4.5 embryo: nuclei and cytoplasm of ICM,  and trophoblast cells. Decidual cells: nuclei  and cytoplasm | Maternally  expressed,  Present  Present    Present    Present  Present    Present | Indirect  immuno-  fluorescence,  RT-PCR | [3] |
| Oocyte, 2-cell to 8-cell embryo, morula;  ED3.5 blastocyst  ED4.5 blastocyst and ICM | Trace or weak  Moderate  Trace or weak | RT-PCT | [8] |
| ED3.5 ICM, trophoectoderm | Strong, present | WISH | [12] |
| ED8.5: diffused expression pattern | Present | In situ hybridization, LacZ staining of gene-trap embryos | [13] |
| ED10.5: central nervous sytem | Ambiguous | RNA in situ | [9] |
| ED12.5: embryo: developing jaw,  presumptive tooth regions | Present | In situ  hybridization | [14] |
| ED13.5: embryo; head, central nervous  system, cerebral cortex, corpus striatum,  thalamus, hypothalamus, midbrain,  hindbrain, ventricular layer, spinal cord, | Present | RNA in situ | [9] |
| P0: head, central nervous system, cerebral  cortex, cerebellum, corpus striatum,  hindbrain, spinal cord, cranial ganglion,  dorsal root ganglion, tooth. | Present | RNA in situ | [9] |

**References**
