# Supplementary material for: Expression of the transcription factor, TFII-I, during post-implantation mouse embryonic development
Source: BMC Res Notes. 2010 Jul 20;3:203. doi: 10.1186/1756-0500-3-203 (PMC2921380; doi:10.1186/1756-0500-3-203)
Supplement: Additional file 4 — Methods. [file 1756-0500-3-203-S4.DOC]

***Expression of the transcription factor, TFII-I, during post-implantation mouse embryonic development****,* Fijalkowska I, Sharma D, Bult JC, Danoff SK

**Additional File 4: Methods**

All animal work was approved by the Johns Hopkins Animal Care and Use Committee and conforms with internationally recognized guidelines.

TFII-I antibody (Ab 3588) was generated by injecting synthetic peptide ((151)QGLPEGVAFKHPE) conjugated with hemaglutinin, as previously described [1]. The polyclonal Ab 3588 is a pan-specific antibody which recognizes all isoforms of TFII-I. Rabbit serum was used as a source of TFII-I primary antibody. Serum pre-incubated with the peptide was used as a control for non-specific staining. TFII-IRD1 antibody was generated as previously described [1]. Mouse post-implantation embryos were obtained from Novagen. All sections were blocked in normal goat serum (NGS) (Vector, Burlingame, CA) (3:200) in PBS for 40 minutes at room temperature. The sections were then incubated overnight at 4°C with TFII-I serum diluted 1:750 in Antibody Diluent Reagent Solution (Zymed, S. San Francisco, CA, USA). A goat anti-rabbit biotinylated antibody (1:200) in 1.5% NGS was used as a secondary antibody. Immunohistochemistry was performed using ABC Reagents (Vector) followed by 3'3' – diaminobenzidine (Vector). Sections were counterstained with hematoxylin (Vector) as recommended by the manufacturer. Sections were visualized using a Zeiss Axiovert S100TV microscope and Metamorph 4.5.6 software (Universal Imaging, Dowington, PA, USA).

References

1. SK Danoff, HE Taylor, S Blackshaw, S Desiderio: **TFII-I, a candidate gene for Williams syndrome cognitive profile: parallels between regional expression in mouse brain and human phenotype**. *Neuroscience* 2004, **123**:931-8.
